# Exploring the contribution of straw utilization to carbon emission reduction in Anhui Province (China)

**DOI:** 10.1371/journal.pone.0349747

**Published:** 2026-05-27

**Authors:** Zhou Ye, Xiaohang Yu, Ruoyun Yao, Youzhi Yao

**Affiliations:** College of Materials Engineering, Wuhu Vocational Technical University, Wuhu, China; University of Minnesota, UNITED STATES OF AMERICA

## Abstract

Taking various prefecture-level cities in Anhui Province as the subject of this study, this research draws on data from the ‘Anhui Statistical Yearbook’ to analyze crop straw resources’ potential full utilization, and spatial distribution characteristics, in Anhui Province for the year 2023. The carbon neutralizing effect of straw full utilization was also evaluated using life cycle assessment. Results indicate that the total theoretical straw resources from major crops in Anhui Province in 2023 amounted to 5.213 × 10^7^ tons (t), dominated by wheat, rice, and corn straw; which collectively accounted for 89.72% of the total. The carbon emission reductions from straw utilization through fertilization, animal feed, energy generation, substrate application, and raw material processing were approximately 4 × 10^6^, 1.07 × 10^6^, 5.8 × 10^5^, 9.8 × 10^4^, and 1.67 × 10^5^ t of CO₂, respectively. Clarifying the total amount, types, potential utilization, and spatial distribution of straw resources at the city level is essential for promoting rational resource allocation and facilitating logicall regional planning for the utilization of those resources. These findings are of paramount importance towards efforts to achieve the goals of “Carbon peaking and carbon neutrality” (Dual-carbon) and fostering coordinated economic and social development in China. Under the framework of “dual carbon” national strategy and the overall layout of agricultural carbon emission reduction, the data analysis results of straw resource utilization in Anhui Province can serve as a reference for other regions to carry out relevant work.

## 1. Introduction

The goal of Carbon peaking and carbon neutrality (“Dual-carbon”), as the core strategy of Chinese ecological civilization construction, is not only a solemn commitment to address global climate change, but also a key lever for promoting green socio-economic transformation. In 2021, the Chinese government explicitly proposed to “build a clean, low-carbon, safe and efficient energy system, and accelerate innovation in agricultural green development models” [1]. The agriculture, forestry and other land use (AFOLU) sector even contributed 22% of global anthropogenic net greenhouse gas emissions in 2019, becoming an important key area for China to achieve the “dual carbon” goals [2]. Agriculture, as an important sector of carbon emissions, accounts for about 15% of the total national carbon emissions in terms of greenhouse gases, while also possessing dual attributes of carbon source and sink [3–6]. Greenhouse gases (such as methane and nitrous oxide) released from agricultural production interact with the soil’s carbon sequestration potential, which can both contribute to increased greenhouse gas emissions and enhance carbon sequestration potential through soil management and straw returning [7]. In this context, promoting the resource utilization of agricultural waste (e.g., straw) serves as a critical link between the “Dual-carbon” goals and the circular economy. On one hand, converting waste into bioenergy or soil amendments can reduce dependence on fossil fuels, enhance carbon sinks, and directly contribute to emission reductions. On the other hand, it embodies the principles of the circular economy by creating value from by-products.

Straw is the main waste in agricultural production, containing elements such as nitrogen, phosphorus, potassium, and calcium; as well as organic matter such as cellulose and hemicellulose. It has the property of carbon neutrality and has the potential for multiple utilization pathways [8]. Due to factors such as seasonality, dispersion, the unwillingness of growers to collect it, and the high costs associated with its collection, the utilization effect of straw resources is not satisfactory. The indiscriminate disposal of, and open burning of, straw has been repeatedly banned; as these practices contribute significantly to aggregate carbon emissions (Burning a ton of straw releases approximately 1.5 t of CO_2_ eq·t^-1^ tons), this exacerbates haze pollution (accounting for 20%−30% of the regional PM2.5 contribution) thereby threatening ecological security and public health. The continuous mechanization of straw returning to the field annually has exceeded the soil’s carrying capacity and increased the risk of pests and diseases in sunsequent crops [9–11]. Therefore, improper disposal of straw not only causes waste of straw resources, but also has an adverse impact on the ecological environment [12]. To address the environmental and carbon emission problems caused by open straw burning, China has gradually established a policy system from local control to nationwide strict governance. The implementation stages of the ban on open straw burning can be divided into the incidental control stage before 2010, the policy improvement stage from 2010 to 2013, and the nationwide high-intensity implementation stage after the issuance of the “Action Plan for Air Pollution Prevention and Control” in 2013. Driven by national policies, remarkable achievements have been made: the fire carbon emissions from open burning of wheat straw in the North China Plain alone decreased by 3 Mt CO_2_-eq a^-1^ from 2017 to 2021 compared with 1997–2016, while the global carbon emissions from biomass burning showed an upward trend during the same period [13], demonstrating the effectiveness of China’s agricultural carbon emission control policies.Straw is beneficial when utilized, but harmful when discarded [14]. Existing research on straw utilization has primarily concentrated on its methods, namely the “Five-transformations” utilization mode of “fertilization, feed conversion, energy conversion, base materials, and raw materials” [15]. The efficient resource utilization of straw is not only an urgent need for pollution reduction and carbon reduction in the agricultural field, but also one of the necessary ways to achieve the “Dual-carbon” goal.

Domestic and foreign scholars have conducted extensive research on the environmental benefits of straw resource utilization. Life cycle assessment (LCA), as an internationally recognized method for quantifying environmental impacts, is used to evaluate the carbon footprint and resource efficiency of pathways such as straw returning, energy conversion (such as biomass power generation), and material conversion (such as sheet manufacturing) [16]. For example, as the largest renewable energy source in the European Union-biomass energy, 14% of it comes from agricultural raw materials [17]. According to reports, 4.95 × 10^8^ t of straw can produce 8.225 × 10^10^ m^3^ of biomethane [18]. However, existing research mostly focuses on the evaluation of a single technological path, lacking systematic analysis of regional and multi scenario scenarios, especially the potential of straw resource utilization and carbon sequestration and emission reduction using LCA methods at the city scale has not been reported. How to construct a “Dual-carbon” oriented multi-level utilization model for straw based on regional resource endowment has become an urgent problem to be solved [19].

This work took Anhui Province as an example, using the “Anhui Statistical Yearbook” and authoritative documents published by various ministries and commissions in China. Based on the analysis of the quantity, composition, spatial distribution, and demand for fully quantified utilization of straw resources in various prefecture level cities in Anhui Province in 2023 [20], the article focuses on the carbon reduction emissions, carbon neutrality intensity, and the potential for carbon sequestration and emission reduction throughout the entire life cycle of straw utilization. The aim is to reveal the “Dual-carbon” synergistic efficiency mechanism of straw utilization, provide scientific basis for Anhui Province to formulate differentiated straw management policies, promote agricultural green and low-carbon transformation, and also provide reference paradigms for optimizing the path of agricultural waste resource utilization in similar regions, and it provides practical cases for the regional implementation of the national agricultural carbon emission reduction strategy.

## 2. Data sources

### 2.1. Research area

Anhui Province belongs to the transitional monsoon climate between warm temperate and subtropical regions, with distinct four seasons and abundant precipitation but uneven spatial and temporal distribution, with an average temperature of around 18 ℃. The total land area of the province is about 1.401 × 10^5^ km^2^. According to the “Anhui Provincial Land Spatial Planning (2021–2035),” the cultivated land ownership is ≥ 5.41 × 10^6^ hm^2^. The layout of crops in Anhui Province presents a pattern of “northern wheat and southern rice, oil cotton along the Yangtze River, and tea and fruit in mountainous areas.” In 2022, the total planting area of crops in the province was about 9.334 × 10^6^ hm^2^, including 7.314 × 10^6^ hm^2^ of grain crops. The economic crops include 5.618 × 10^5^ hm^2^ of oilseeds (70% of which are rapeseed), 1.025 × 10^5^ hm^2^ of vegetables, and 9.89 × 10^4^ hm^2^ of fruits, which belong to a typical sustainable agricultural optimization development zone [21].

### 2.2. Data sources

The basic data for this study comes from the main crop yields, partitioned cultivated land areas, and collectable straw resources published in the 2023 Anhui Statistical Yearbook [20], mainly including seven major crops such as rice plant, wheat, corn, legume, tubers, oilseeds, and cotton. The grain-straw ratio and collectable coefficient are sourced from the straw resource ledger system of the Ministry of Agriculture and Rural Affairs [22], as shown in Table 1.

**Table 1:** Grain-straw ratio and Collectible Coefficient of Major Crops in Anhui Province.

Major Crops	Grain-straw ratio	Collectable coefficient
**Rice plant**	0.84	0.77
**Wheat**	1.14	0.85
**Corn**	1.00	0.91
**Legume**	1.47	0. 56
**Tubers**	0. 46	0. 73
**Oilseeds**	1. 22	0. 83
**Cotton**	2.75	0.94

## 3. Research methods

### 3.1. Estimation of theoretical resource quantity of straw

The theoretical resource quantity of straw refers to the total amount of stem and leaf agricultural by-products remaining after harvest, excluding the edible parts, and does not include the resource quantity of later precision processing and crop roots. It is closely related to the economic yield of straw and the grain-straw ratio [23]. The theoretical resource quantity of straw is determined by the product of crop yield and the grain-straw ratio coefficient, and its specific expression formula is shown in (1):


$$\begin{array}{c} {\text{S}}_{\text{R}}\;={\text{S}}_{\text{EQ}}\;\times \;{\text{S}}_{\text{G}} \end{array}$$ (1)

In the formula: S_R_ is the theoretical resource quantity of crop straw (t), S_EQ_ is the economic yield of crops (t), S_G_ is the ratio coefficient of grain-straw.

### 3.2. Estimation of collectable resources of crop straw

The amount of straw that can be collected refers to the maximum amount of straw resources that can be collected from the field through mechanical and manual harvesting techniques, and can be used by humans. The calculation formula for the amount of crop straw that can be collected is as follows:


$$\begin{array}{c} {\text{S}}_{\text{P}}={\text{S}}_{\text{R}}\;\times {\text{ S}}_{\text{C}} \end{array}$$ (2)

In the formula, S_P_ represents the amount of straw that can be collected (t), S_C_ represents the coefficient of straw that can be collected.

### 3.3. Estimation of Carbon Sequestration and Emission Reduction through Fully Quantitative Utilization of Straw

The fully quantified utilization of straw for carbon reduction refers to the reduction of CO_2_ emissions during the resource utilization of straw, including CO_2_ emissions caused by replacing open burning, CO_2_ emissions throughout the life-cycle of straw substitutes, carbon sinks caused by straw resource utilization, and CO_2_ emissions throughout the entire life-cycle process. The carbon emissions caused by open-air burning of straw are 0. 802 t CO_2_ eq·t^-1^ [24,25], other parameters used in the calculation process are shown in Tables 2 and 3.

**Table 2:** Carbon sink and carbon emission coefficients of straw full quantification utilization process [4,26–28].

Utilization ways of straw		CE_*i*_	Category (to the field)	CS_*i*_
**Fertilizer** **(to the field)**	Root stubble returning	0. 004	Returning straw	0. 147
	Crushing and covering	0. 011		
	-	Returning to the field		
	Deep plowing and returning	0. 028		
	Rotary tillage returning	0. 021		
**Feed**	–	0. 074	Returning manure	0. 132
**Energy**	briquette fuels	0. 090	–	–
	Bundle heating	0. 042	–	–
	fuel ethanol	0. 131	–	–
	biogas engineering	0. 139	Returning biogas residue	0. 513
	gasification	0. 146	Returning charcoal	0. 804
**Basic material**	–	0. 141	Returning bacterial residue	0. 598
**Raw material**	Artificial plate	−0. 362	Reduce logging	0. 308
	papermaking	0. 711		0. 290

**Note:** The accounting boundary of CE_*i*_ is the CO_2_ emissions from the entire process of straw harvesting, returning to the field, harvesting, storage, transportation, processing, transformation, and utilization.

**Table 3:** Substitution coefficients of straw substitutes and their carbon emission coefficients throughout their entire lifecycle [29–34].

Substitutes		ri	CR_*i*_/(t CO₂ eq·t^-^¹)
**Nitrogenous fertilizer**		0.007	0.577
**Phosphate fertilizer**		0.002	0.173
**Potash fertilizer**		0.001	0.049
**Concentrated feed**		0.300	0.192
**Sawdust**		1.000	0.144
**Traditional wooden board**		1.200	0.283
**Traditional paper**		2.358	1.125
**Briquette fuels**	Replace standard coal	0.598	2.500
**Bundle straw**		0.647	
**Biogas**		0.256	
**Pyrolysis gasification gas**		0.182	
**Fuel ethanol**	Replace gasoline	0.124	3.088

**Note:** The accounting boundary of CR_*i*_ is the CO_2_ emissions from the entire process of raw material extraction, processing, manufacturing, use, maintenance, and final disposal of substitutes.


$$\begin{array}{c} \;{\text{CQ}}_{i}=\;({\text{CS}}_{i}\;-{\text{CE}}_{i}\;+\;{\text{CR}}_{i}\;\times \;{\text{r}}_{i})\;\times {\text{SU}}_{i} \end{array}$$ (3)

In the formula, CQ_*i*_ is the amount of CO_2_ that can be reduced when straw is utilized in the i-th way (t CO_2_ eq), CS_*i*_ is the carbon sink coefficient (t CO_2_ eq·t ^−1^) for the i-th utilization of crop straw, CE_*i*_ is the carbon emission coefficient (t CO_2_ eq·t ^−1^) for the entire lifecycle of crop straw when utilized in the i-th way, CR_*i*_ is the carbon emission coefficient (t CO_2_ eq·t ^−1^) of the entire life-cycle of the substitute used for the i-th method of utilizing crop straw, r_*i*_ is the straw substitution coefficient, SU_*i*_ is the quantity (t) of crop straw utilized in the i-th way.

### 3.4. Estimation of carbon neutrality intensity in the full quantitative utilization of straw

The carbon neutrality intensity of fully quantified utilization of straw refers to the amount of CO_2_ that can be neutralized per unit area when crop straw is used for fertilizer, feed, and energy utilization. The larger the value, the stronger the carbon neutrality ability of the region. The specific calculation formula is as follows:


$$\begin{array}{c} \text{CNI}={\text{CQ}}_{i}/\text{A} \end{array}$$ (4)

In the formula, CNI represents the carbon neutrality intensity of crop straw resource utilization potential (t CO_2_ eq·hm^-2^); A is the area of the region (hm^2^).

## 4. Results and analysis

### 4.1. Main crop straw resources and composition in Anhui Province

**Fig 1:**
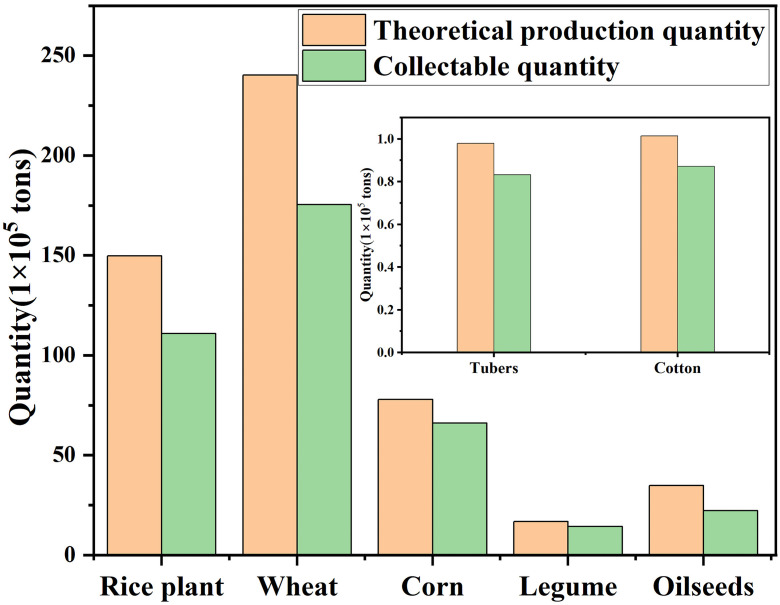
Theoretical and Collectable Quantity of Straw in the Province.

According to the 2023 Anhui Provincial Statistical Yearbook, the theoretical resource quantity of crop straw was calculated using formula (1) based on the crop yields of various types in each city, as shown in Table 4. The comparison between the collectable amount of straw and the collectable resource quantity calculated using formula (2) is shown in Fig 1. The theoretical resource quantity of straw in the province is about 5.213 × 10^7^ t, with a collection rate of 93.1%. Among them, the proportion of cereal straw is nearly 90%, wheat straw is about 2.402 × 10^7^ t, rice straw is about 1.497 × 10^6^ t and corn straw is about 7.781 × 10^6^ t. These three contribute a total of about 4.677 × 10^7^ t, which can provide a solid raw material foundation for soil organic matter improvement, feed processing, and biomass energy conversion. At the same time, there are about 3.477 × 10^6^ t of oil crop straw, 167.9 t of legume straw, and about 2.0 × 10^5^ t tons of other crops such as potatoes and cotton and hemp, totaling about 5.356 × 10^6^ tons, providing diversified utilization possibilities for biobased boards, composite materials, and fermentation production.

**Table 4:** Theoretical resource quantity data of various crop straw in Anhui Province.

Region	Total (tons)	Grain (tons)					Oilseeds (tons)	Cotton (tons)
		Cereals			Legume	Tubers		
		Rice plant	Wheat	Corn				
**Hefei**	3446655	2069296	844720	119170	36913	6698	364033	5825
**Huaibei**	2049735	-	1422483	480774	135055	433	10979	10
**Bozhou**	6885884	19503	4462443	1967838	314340	8043	113438	280
**Suzhou**	6285961	7552	3868848	1765752	361613	22884	259241	70
**Bengbu**	4136530	584109	2146114	748772	64617	1881	591036	2
**Fuyang**	7113292	318324	4575437	1686697	381346	13832	137176	480
**Huainan**	3668325	1767016	1690707	71345	55082	2049	82127	-
**Chuzhou**	5642655	2421606	2573827	252456	111539	6645	276438	145
**Lu’an**	4026723	2498090	1033431	176836	46011	3766	265514	3075
**Maanshan**	1283752	740170	357646	15398	11960	4248	153458	870
**Wuhu**	1660648	1040751	298310	71501	21170	3257	212294	13365
**Xuancheng**	1458579	923182	310751	48282	21836	5747	148781	-
**Tongling**	720213	456500	80378	31026	12257	1087	131426	7540
**Chizhou**	839153	511314	82063	53103	21486	2275	165427	3485
**Anqing**	2548713	1431972	274712	201336	64892	9891	499878	66030
**Huangshan**	363571	181759	-	90838	19487	5193	66144	150
**Total**	5213088	1497143	24021871	7781125	1679605	97930	3477388	101327

**Fig 2:**
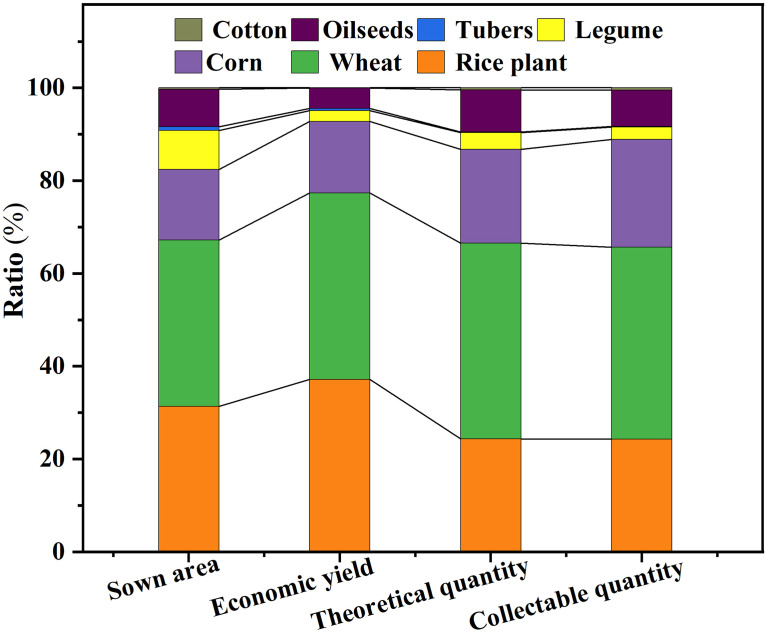
Sowing area, yield, and proportion of straw resources of major crops in Anhui Province in 2023.

The planting area, yield, and proportion of straw resources of major crops in Anhui Province in 2023 are shown in Fig 2. It can be seen that the straw resources in Anhui Province present a pattern of “grain dominated, non grain supplemented” distribution, with advantages in the northern wheat belt, rice concentration area, and northern Anhui corn area. The southern oilseed area and scattered bean area have promising potential for deep processing and refined utilization. This resource structure not only provides a quantitative basis for the path selection of straw returning, feed utilization, and energy utilization under the background of “carbon peak and carbon neutrality,” but also lays a solid data foundation for the formulation of unified planning and regional differentiated policies throughout the province.

### 4.2. Concentration and spatial distribution of collectable resources of main straw in Anhui Province

**Fig 3:**
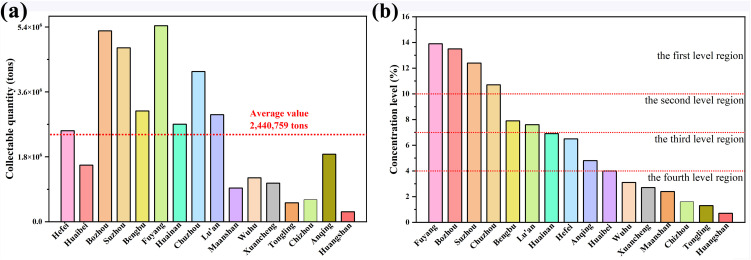
Straw collection situation (a) collectable resource quantity and average value in each city, and (b) concentration level.

In 2023, the regional average of collectable straw resources in Anhui Province will be 2440759 t, as shown in Fig 3a. Among them, Fuyang has the largest collectable crop straw resources of 5433427 t, and Huangshan has the smallest collectable crop straw resources of 275152 t, which is only 11.27% of the average level; Among the 16 cities, 8 cities including Hefei, Bozhou, Suzhou, Bengbu, Fuyang, Huainan, Chuzhou, and Lu’an have straw collectible resources that exceed the regional average, while others have not reached the regional average level.

The degree of concentration can be determined by the proportion of straw resources that can be collected in each city to the total area. The ratio multiplied by 100 within 10–14 is the first level region, within 7–10 is the second level region, within 4–7 is the third level region, and within 0–4 is the fourth level region. As shown in Fig 3b, there are four first districts for the collection of crop straw resources, namely Bozhou, Suzhou, Fuyang, and Chuzhou, and two secondary districts, namely Bengbu and Lu’an. There are four third level districts, namely Hefei, Huaibei, Huainan, and Anqing. There are six fourth level districts, namely Ma’anshan, Wuhu, Xuancheng, Tongling, Chizhou and Huangshan, which is mainly due to the fact that the land and planting area of these six cities are less than those of other cities.

In terms of spatial distribution, high potential areas are mainly distributed in the Bozhou-Suzhou-Fuyang plain area of the northern Anhui, with good soil fertility, large crop planting areas, and high mechanization, which concentrates the potential for straw returning to the field. The Bengbu-Wuhu-Chuzhou district in central and eastern Anhui has also shown strong ability to recycle farmland resources. Huangshan, Chizhou, Xuancheng and Tongling, which are due to limited arable land and a high proportion of forestry and fruit industry, the amount of straw that can be collected in the mountainous and hilly areas of southern Anhui is relatively low, forming a low potential area.

### 4.3. Analysis of full quantitative utilization of straw

In recent years, through policy guidance and technological innovation, Anhui has formed a “diversified and high-value” development path in the field of straw comprehensive utilization. According to the “Technical Guidelines for Total Treatment and Utilization of Regional Crop Straw” released by the Ministry of Agriculture, the recommended range for straw return in the middle and lower reaches of the Yangtze River is 3–9 t/hm^2^. Calculated based on a suitable return amount of 4.5 t/hm^2^, the required amount of straw for fertilizer utilization is 4.20 × 10^7^ t, accounting for 78.06% of the collectable straw resources and the highest proportion of straw resource utilization. Based on the cultivated land area of each city, the spatial distribution characteristics of the demand for straw fertilization in Anhui Province were estimated. Fuyang has the highest demand for straw fertilization, followed by Bozhou and Chuzhou, mainly due to its relatively large cultivated land area.

The fertilizer utilization mainly adopts the “crushing and returning to the field technology” to treat straw. The technical standard requires a crushing length of ≤ 10 cm, combined with deep plowing of 20 cm, and the addition of fast rot agent (30 kg/hm^2^) and organic fertilizer (900 kg/hm^2^) to achieve rapid improvement of soil organic matter. It is also possible to convert discarded straw into high value-added commercial fertilizers through the production of straw charcoal based fertilizers and bacterial residue organic fertilizers.

The main challenge facing fertilizer utilization is balancing the cost of returning farmland with long-term effects. In response, Anhui Province has increased farmers’ participation and ensured the implementation of technology through a monitoring network for farmland quality and subsidies for deep plowing.

Feed utilization is a successful example of high-value transformation of straw in Anhui Province. In 2023, the conversion volume of the “straw-to-feed” project in the province will exceed 6 × 10^6^ t, accounting for more than 16% of the total straw utilization in the province, and driving the formation of a 12 billion yuan output value industrial chain. According to the “Technical Guidelines for Total Treatment and Utilization of Regional Crop Straw,” different animal husbandry unit coefficients and straw consumption, as well as the year-end inventory of herbivorous livestock in each city, the straw consumption of sheep is 0.6 t/one/year, and pigs and cattle are calculated as 5 times that of sheep. The spatial distribution characteristics of the demand for straw feed in Anhui Province were estimated. The regions with high demand for straw feed are mainly concentrated in the northern part of Anhui Province, with Fuyang City having the largest demand for straw feed, far greater than other cities. This is the result of policy driven and scale effects.

Energy utilization is the supporting path for the transition to clean energy. Huainan, which has the highest proportion of demand, as an example, energy utilization accounts for 28.4% of the straw consumption structure, mainly through technologies such as biomass power generation, solidified fuel, and pyrolysis gasification to achieve straw resource utilization. Small and mobile devices are rapidly being promoted in rural clean energy projects. Fuyang is piloting the “Straw-Gasification-Clean-Energy” project, where a single village’s gas supply system consumes 500 t of straw annually, meeting the energy needs of 200 households for cooking. This type of project reduces the use of loose coal and improves indoor air quality by replacing wages with gas, with both environmental and health benefits.

Although the utilization of basic materials accounts for a relatively small proportion in the “Five-transformations,” it has significant potential for value enhancement. Anhui Province mainly develops edible mushroom cultivation and seedling substrate. According to statistical yearbook data, the proportion of straw based materials is less than 5%. In 2023, the total amount of edible mushrooms in Anhui Province was 7.542 × 10^5^ t, a year-on-year increase of 6.55%, the total demand for straw was 4.525 × 10^6^ t. With the development of economy, Yixian of Huangshan, Dongzhi of Chizhou and other places have formed distinctive industrial clusters of edible fungi. The planting area of edible fungi in Dongzhi County is 460 hm^2^, and the output is nearly 1.3 × 10^5^ t. The bottleneck of the development of straw based materials lies in “technical standardization” and “market stability,” which leads to a small proportion of straw based material practice. Therefore, local governments have introduced different policies to improve the utilization rate of straw based materials. For example, Hefei has set up special incentive funds to provide equipment renewal subsidies to enterprises that consume more than 500 t of straw annually, accelerating technological penetration.

Anhui straw raw material focuses on the development and utilization of material properties, mainly covering three directions: environmental protection, biobased, and packaging materials. Several enterprises in Anhui have developed products such as biomass degradation films and straw eco-friendly tableware, with technology sourced from research institutions such as the University of Science and Technology of China and Hefei University of Technology. Bengbu has introduced the technology of “straw fiber mulching film” to effectively solve the problem of residual film pollution in farmland. The challenge of raw material utilization lies in “cost competitiveness” and “market acceptance” for theoretical demand in various regions.

**Fig 4:**
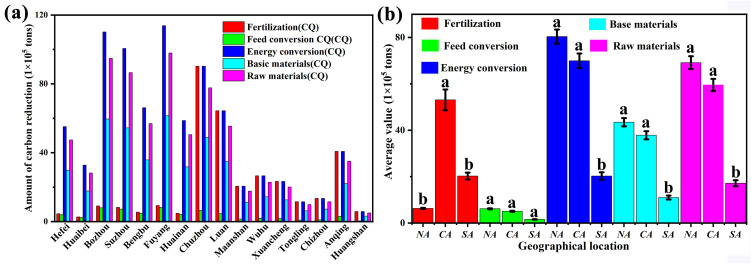
Analysis of Carbon Emission Reduction (a) Carbon emission reduction from five-transformations in various regions (b) Geographical variation in resource utilization patterns.

According to the calculation formula and method mentioned above, the carbon emission reductions of different utilization methods are calculated, as shown in Fig 4a. According to formula (3), the “Five-transformations” of straw reduce approximately 5.915 × 10^6^ t of CO_2_ emissions, while the unused straw generates approximately 9.50 × 10^6^ t of CO_2_ emissions. By subdividing the “Five-transformations” of straw utilization, the specific carbon emission reductions for straw fertilization, feed utilization, energy utilization, base material utilization, and raw material utilization in Anhui Province in 2023 are calculated to be approximately 4.00 × 10^6^, 1.07 × 10^6^, 5.80 × 10^5^, 9.80 × 10^4^, and 1.67 × 10^5^ t of CO_2_, accounting for 67.57%, 18.10%, 9.80%, 1.68%, and 2.85%, respectively. Among them, the use of fertilizers and feed has the largest contribution to emission reduction, accounting for a total of 88.67% of the total emission reduction. Energy conversion follows closely, while the use of base materials and raw materials has the lowest emission reduction, accounting for only 4.53% of the total.

Geographically, the 16 cities of Anhui Province are typically divided into three regions: Northern Anhui (NA) (Suzhou, Huaibei, Bengbu, Fuyang, Huainan, Bozhou), Central Anhui (CA) (Hefei, Lu’an, Chuzhou), and Southern Anhui (SA) (Huangshan, Chizhou, Xuancheng, Ma’anshan, Wuhu, Tongling, Anqing). One-way analysis of variance (ANOVA) was conducted to examine the mean differences in the five resource utilization patterns among the three groups, with the Kruskal-Wallis nonparametric test used for verification. For indicators with significant ANOVA results, the Tukey’s honestly significant difference (HSD) post-hoc test was performed, and subgroups with no significant differences were labeled with lowercase letters (a, b), where distinct letters indicate statistically significant differences at the 0.05 level (Fig 4b).

One-way ANOVA revealed significant regional differences for fertilization (F = 7.028, p = 0.008), energy conversion (F = 12.24, p = 0.001), base material utilization (F = 12.24, p = 0.001), and raw material utilization (F = 12.24, p = 0.001). No significant difference was found for feed utilization (F = 3.012, p = 0.083). The Kruskal-Wallis test corroborated these results, showing significant differences for all but feed utilization (p < 0.05).

Tukey’s HSD post-hoc test (α = 0.05) indicated that for fertilization, the mean value in Central Anhui (53.084 × 10^5^ t) was significantly higher than in Northern (6.304 × 10^5^ t) and Southern Anhui (20.257 × 10^5^ t), while the latter two did not differ significantly (labeling: a, b, b). For energy conversion, base material, and raw material utilization, both Northern Anhui (80.37 × 10^5^ t, 43.49 × 10^5^ t, 69.17 × 10^5^ t) and Central Anhui (69.95 × 10^5^ t, 37.84 × 10^5^ t, 60.18 × 10^5^ t) were significantly higher than Southern Anhui (20.28 × 10^5^ t, 10.97 × 10^5^ t, 17.45 × 10^5^ t), with no significant difference between Northern and Central Anhui. Consequently, Northern and Central Anhui share the label “a”, while Southern Anhui is labeled “b” (i.e., a, a, b). Feed utilization showed no significant regional differences, with all regions labeled “a”.

### 4.4. Analysis of carbon neutrality intensity characteristics of straw resource utilization

In the scenario calculation of 5.213 × 10^7^ t of collectable straw in the province for returning to the field in 2023, the carbon neutrality intensity (CNI) of the fertilization pathway in Anhui Province showed significant band like differentiation in space, and the differences between different cities were quite significant. The average CNI in the province is about 0.065 t CO_2_ eq/hm^2^, but the actual values in each city range from 0.050 t CO_2_ eq/hm^2^ to 0.080 t CO₂ eq/hm^2^. Chuzhou leads the way with a highest level of 0.078 t CO₂ eq/hm^2^, mainly due to the dual advantages of highly concentrated arable land and a mechanization rate of 92% for straw returning Ma’anshan City closely follows, with a CNI of 0.075 t CO₂ eq/hm^2^. The combination of rice rapeseed rotation mode and deep tillage technology in the plow layer significantly increases the soil organic carbon sequestration efficiency of straw compared to other areas in the province. The cities of Hefei and Wuhu along the Yangtze River have achieved a carbon neutrality intensity level of approximately 0.070 t CO₂ eq/hm^2^, attributed to their high soil fertility and well-equipped machinery for returning farmland, demonstrating the advantages of the fertilization pathway in plain areas. In contrast, although the amount of straw returned to the fields in Bozhou and Fuyang in the northern Anhui Plain reached 3.85 × 10^6^ tons and 4.2 × 10^6^ tons respectively, their carbon sequestration efficiency per unit soil area was only 0.055–0.060 t CO₂ eq/hm^2^ due to their larger arable land area base; In Huangshan, Chizhou and Xuancheng in southern Anhui, due to the undulating terrain and scattered cultivated land, the CNI is mostly lower than 0.050 t CO₂ eq/hm^2^.

## 5. Discussions

1) Potential for carbon sequestration through fertilization. The main ways of utilizing straw fertilizers include technologies such as root stubble returning, crushing and covering returning, deep plowing returning, and rotary tillage returning. Meta analysis was conducted on the impact of continuous straw returning on soil organic carbon in Chinese farmland. Compared with no straw returning, straw returning significantly increased soil organic carbon content, with an average increase of 13.97 ± 1.38% [35–37]. Based on literature research [38–40], assuming that the proportion of straw returning organic carbon fixed in the soil is 10% and the total organic carbon content of straw is 0 The calculated soil carbon sink for returning straw to the field is 146. 8 g CO_2_ eq/kg.
2) Potential for carbon sequestration through feed utilization. The main way of utilizing straw as feed is through dry straw and coarse feed, and the study does not currently consider whole plant silage feed for corn. Research has shown that the total organic carbon storage in plots treated with nitrogen, phosphorus, potassium, and farmyard manure increased by 25% and 45%, respectively, compared to plots treated with nitrogen, phosphorus, potassium, and fallow fields [41]. According to studies by different researchers, the carbon sequestration effect of returning livestock and poultry manure to the field is better than directly returning straw, with an organic carbon sequestration rate of 20% to 30% [42]. According to the IPCC guidelines (2006), the digestion rate of ruminants is 55%; The carbon sequestration rate of manure organic carbon in farmland is calculated at 20%, and the indirect carbon sink for straw feed utilization is 132.0 g CO_2_ eq/kg.
3) Energy based carbon sequestration potential. The energy utilization of straw mainly includes technologies such as molded fuel, bundled heating, biogas/biogas, co production of pyrolysis carbon and gas, direct combustion of straw for power generation, and fuel ethanol. Alternative fossil fuels are calculated by offsetting the heat of coal (equivalent to standard coal), based on the unit calorific value of raw coal with a carbon content of 26.37 g /MJ, the carbon oxidation rate is 0.94. Based on the 2006 IPCC Guidelines for National Greenhouse Gas Inventories and referring to the relevant calculation methods for coal emission factors in the Guidelines for the Calculation Methods and Reporting of Greenhouse Gas Emissions of Chinese Power Generation Enterprises (Trial), there are certain differences in greenhouse gas emissions from different straw utilization technologies, and the amount of carbon sequestration in soil as a by-product of large-scale biogas/bio natural gas and pyrolysis carbon gas co production technologies is 513. 4, 803. 5 g CO_2_ eq/kg.
4) Carbon sequestration potential of straw based material conversion. The main utilization methods of straw based composting are edible mushroom cultivation and waste mushroom residue composting returning to the field. Related studies have shown that when using straw and soybean straw as partial substitutes for sawdust from broad-leaved trees to cultivate black fungus, with a straw substitution ratio of 25% to 35%, the growth of mycelium and fruiting bodies is basically the same as that of sawdust cultivation from broad-leaved trees [43]. Therefore, the use of straw as a base material can replace some forest resources. According to calculations, the forest carbon sequestration capacity of straw based material utilization as a substitute for forest resources is 462 g CO_2_ eq/kg. The carbon loss rate during the mushroom production process is 30% [44]. Assuming that the organic carbon fixation rate of mushroom in farmland is equal to the carbon fixation rate of straw returning, and calculated at 10%, the soil carbon sink of waste mushroom residue returning to farmland is 136.1 g CO_2_ eq/kg.
5) Raw material carbon sequestration potential. The raw material utilization of straw is mainly focused on artificial boards and papermaking. Currently, the final treatment of waste straw artificial boards or straw paper is mainly used for combustion power generation. The consumption of raw wood for artificial boards is 1. 1 m^3^ /m^3^, 1 kg of artificial board consumes 1. 5 kg, The density of artificial board is 650 kg/m^3^ [45], and the amount of forest carbon sink generated by using straw artificial board as a raw material to replace wood logging is 308 g CO_2_ eq/kg.

Although the unit straw raw material utilization has the highest carbon sequestration and emission reduction capacity, the current amount of raw material utilization is the least. Governments at all levels should introduce corresponding policies to encourage and support the construction of a group of manufacturers that use crop straw to produce artificial boards and paper, and increase the raw material utilization rate of straw. The carbon neutrality intensity of energy utilization of crop straw in urban areas is the highest, at 0. 2–0. 7 t CO_2_ eq·hm^-2^, Among the five common energy utilization methods, the carbon sequestration and emission reduction capabilities of preparing formed fuels and bundling heating are relatively high. These two energy utilization methods can be prioritized, but the economic benefits brought by producing fuel ethanol are significantly higher than other methods. Therefore, governments at all levels should also encourage the construction of a number of biomass fuel ethanol enterprises.Huangshan, Chizhou and other places are not suitable for crop planting because of their large mountainous area. In addition to the necessary straw returning, the local government should focus on promoting the energy and raw material utilization of straw.

Based on the above analysis, clarifying the total amount, types, full quantitative utilization potential, and spatial distribution characteristics of straw resources in the city is the basis for resource utilization and scientific planning of straw. At the same time, evaluating the emission reduction and carbon fixation under various resource utilization methods from the perspective of the entire life cycle plays an important role in promoting the achievement of local “Dual-carbon” goals and the coordinated development of the economy and society.

From the perspective of the overall national strategy for agricultural carbon emission reduction, the research on straw resource utilization in Anhui Province also provides regional data support for national-level straw governance. As the world’s largest carbon emitter, China’s agricultural emission reduction is an important component of achieving the 2030 carbon peak and 2060 carbon neutrality goals [46]. As a key measure for agricultural emission reduction, the large-scale and model-based development of regional practices in straw resource utilization will further promote the carbon emission control in the national agricultural AFOLU sector.

## Supporting information

S1 Table Data supporting Fig, 1. Theoretical and Collectable Quantity of Straw in the Province.
(DOCX)

S2 Table Data supporting Fig. 2. Sowing area, yield, and proportion of straw resources of major crops in Anhui Province in 2023.
(DOCX)

S3 Table Data supporting Fig 3. Collectable Resource Quantity and Average of Crop Straw in Each City. (DOCX)

S4 Table Distribution of Demand for Five-transformations in Each City. (DOCX)

S5 Table Data supporting Fig. 4. Analysis of Carbon Emission Reduction (a) Carbon emission reduction from five-transformations in various regions. (b) Geographical variation in resource utilization patterns.
(DOCX)

S6 Table Carbon neutrality intensity (CNI) characteristics of straw resource utilization in various regions. (DOCX)
