# Supplementary material for: Exploring the contribution of straw utilization to carbon emission reduction in Anhui Province (China)
Source: PLoS One. 2026 May 27;21(5):e0349747. doi: 10.1371/journal.pone.0349747 (PMC13215477; doi:10.1371/journal.pone.0349747)
Supplement: S1 Table — Theoretical and Collectable Quantity of Straw in the Province. (DOCX) [file pone.0349747.s001.docx]

**S1 Table Data supporting Fig. 1.**

**Theoretical and Collectable Quantity of Straw in the Province**

|  | **Theoretical resource quantity**  **(tons)** | **Collectable quantity**  **(tons)** |
| --- | --- | --- |
| **Rice plant** | 14971143 | 11,078,646 |
| **Wheat** | 24021871 | 17,535,966 |
| **Corn** | 7781124 | 6,613,956 |
| **Legume** | 1679605 | 1,427,663 |
| **Tubers** | 3477388.4 | 2,225,529 |
| **Oilseeds** | 97930 | 83240 |
| **Cotton** | 101327 | 87141 |
