# Supplementary material for: Exploring the contribution of straw utilization to carbon emission reduction in Anhui Province (China)
Source: PLoS One. 2026 May 27;21(5):e0349747. doi: 10.1371/journal.pone.0349747 (PMC13215477; doi:10.1371/journal.pone.0349747)
Supplement: S2 Table — Sowing area, yield, and proportion of straw resources of major crops in Anhui Province in 2023. (DOCX) [file pone.0349747.s002.docx]

**S2 Table Data supporting Fig. 2.**

**Sowing area, yield, and proportion of straw resources of major crops in Anhui Province in 2023**

|  | **Rice plant** | **Wheat** | **Corn** | **Legume** | **Tubers** | **Oilseeds** | **Cotton** |
| --- | --- | --- | --- | --- | --- | --- | --- |
| **Sown area** | 2500689 | 2862705 | 1209606 | 675307 | 64782 | 643463 | 22658 |
| **Economic yield** | 16098000 | 17407200 | 6650533 | 1036791 | 204019 | 1889883 | 20265 |
| **Theoretical quantity** | 19163100 | 33150200 | 15951300 | 2821735 | 113598 | 7128646 | 336406 |
| **Collectable quantity** | 14180700 | 24199600 | 13558600 | 1580172 | 82927 | 4562333 | 289309 |
