# Supplementary material for: Exploring the contribution of straw utilization to carbon emission reduction in Anhui Province (China)
Source: PLoS One. 2026 May 27;21(5):e0349747. doi: 10.1371/journal.pone.0349747 (PMC13215477; doi:10.1371/journal.pone.0349747)
Supplement: S3 Table — (DOCX) [file pone.0349747.s003.docx]

**S3 Table Data supporting Fig. 3.**

**Collectable Resource Quantity and Average of Crop Straw in Each City**

| **Collectable quantity (tons)** | |  | **Concentration level** | |
| --- | --- | --- | --- | --- |
| Hefei | 2524279 |  | Fuyang | 13.9 |
| Huaibei | 1569271 |  | Bozhou | 13.5 |
| Bozhou | 5291544 |  | Suzhou | 12.4 |
| Suzhou | 4823534 |  | Chuzhou | 10.7 |
| Bengbu | 3070147 |  | Bengbu | 7.9 |
| Fuyang | 5433427 |  | Lu'an | 7.6 |
| Huainan | 2703573 |  | Huainan | 6.9 |
| Chuzhou | 4162970 |  | Hefei | 6.5 |
| Lu'an | 2968186 |  | Anqing | 4.8 |
| Maanshan | 934635 |  | Huaibei | 4 |
| Wuhu | 1216823 |  | Wuhu | 3.1 |
| Xuancheng | 1069708 |  | Xuancheng | 2.7 |
| Tongling | 524797 |  | Maanshan | 2.4 |
| Chizhou | 612483 |  | Chizhou | 1.6 |
| Anqing | 1871609 |  | Tongling | 1.3 |
| Huangshan | 275153 |  | Huangshan | 0.7 |
