# Supplementary material for: Exploring the contribution of straw utilization to carbon emission reduction in Anhui Province (China)
Source: PLoS One. 2026 May 27;21(5):e0349747. doi: 10.1371/journal.pone.0349747 (PMC13215477; doi:10.1371/journal.pone.0349747)
Supplement: S4 Table — (DOCX) [file pone.0349747.s004.docx]

**S4 Table Distribution of Demand for Five-transformations in Each City**

| **City** | **Fertilization CQ**  **(tons)** | **Feed conversion CQ**  **(tons)** | **Energy conversion**  **CQ**  **(tons)** | **Base materials**  **CQ**  **(tons)** | **Raw materials**  **CQ**  **(tons)** |
| --- | --- | --- | --- | --- | --- |
| **Hefei** | 454262.26 | 398433.34 | 5514648.32 | 2983080.08 | 4744031.36 |
| **Huaibei** | 270150.92 | 236949.32 | 3279575.39 | 1774045.31 | 2821287.53 |
| **Bozhou** | 907545.79 | 796008.24 | 11017415.07 | 5959732.97 | 9477841.49 |
| **Suzhou** | 828477.06 | 726657.06 | 10057537.22 | 5440499.04 | 8652096.97 |
| **Bengbu** | 545186.36 | 478182.85 | 6618447.71 | 3580166.56 | 5693585.83 |
| **Fuyang** | 937517.64 | 822296.54 | 11381266.93 | 6156554.08 | 9790848.69 |
| **Huainan** | 483477.91 | 424058.38 | 5869320.1 | 3174935.34 | 5049141.31 |
| **Chuzhou** | 743690.62 | 652290.9 | 9028247.7 | 4883717.74 | 7766640.36 |
| **Luan** | 530714.04 | 465489.18 | 6442756.77 | 3485128.74 | 5542445.94 |
| **Maanshan** | 169195.91 | 148401.7 | 2054002.77 | 1111087.12 | 1766976.42 |
| **Wuhu** | 218870.09 | 191970.91 | 2657036.88 | 1437290.89 | 2285742.55 |
| **Xuancheng** | 192237.83 | 168611.77 | 2333726.88 | 1262400.38 | 2007611.89 |
| **Tongling** | 94922.67 | 83256.66 | 1152341.25 | 623344.59 | 991313.08 |
| **Chizhou** | 110598.71 | 97006.11 | 1342645.07 | 726287.07 | 1155023.85 |
| **Anqing** | 335915.22 | 294631.17 | 4077940.11 | 2205910.73 | 3508088.76 |
| **Huangshan** | 47917.9 | 42028.78 | 581713.23 | 314670.5 | 500424.62 |

|  |  |  |  |  |  |  |  |  |  |
| --- | --- | --- | --- | --- | --- | --- | --- | --- | --- |
|  |  |  |  |  |  |  |  |  |  |
|  |  |  |  |  |  |  |  |  |  |
|  |  |  |  |  |  |  |  |  |  |
|  |  |  |  |  |  |  |  |  |  |
|  |  |  |  |  |  |  |  |  |  |
|  |  |  |  |  |  |  |  |  |  |
|  |  |  |  |  |  |  |  |  |  |
|  |  |  |  |  |  |  |  |  |  |
|  |  |  |  |  |  |  |  |  |  |
|  |  |  |  |  |  |  |  |  |  |
|  |  |  |  |  |  |  |  |  |  |
|  |  |  |  |  |  |  |  |  |  |
|  |  |  |  |  |  |  |  |  |  |
|  |  |  |  |  |  |  |  |  |  |
|  |  |  |  |  |  |  |  |  |  |
|  |  |  |  |  |  |  |  |  |  |
