# Supplementary material for: Exploring the contribution of straw utilization to carbon emission reduction in Anhui Province (China)
Source: PLoS One. 2026 May 27;21(5):e0349747. doi: 10.1371/journal.pone.0349747 (PMC13215477; doi:10.1371/journal.pone.0349747)
Supplement: S5 Table — Analysis of Carbon Emission Reduction (a) Carbon emission reduction from five-transformations in various regions. (b) Geographical variation in resource utilization patterns. (DOCX) [file pone.0349747.s005.docx]

**S5 Table Data supporting Fig. 4.**

**Analysis of Carbon Emission Reduction Carbon emission reduction from five-transformations in various regions**

**Geographical variation in resource utilization patterns**

| **City** | **Geographical Regions** | **Fertilization** | **Feed conversion** | **Energy conversion** | **Base materials** | **Raw materials** |
| --- | --- | --- | --- | --- | --- | --- |
| **Suzhou** | NA | 8.28477 | 7.26657 | 100.57537 | 54.40499 | 86.52097 |
| **Huaibei** | NA | 2.70151 | 2.36949 | 32.79575 | 17.74045 | 28.21288 |
| **Bengbu** | NA | 5.45186 | 4.78183 | 66.18448 | 35.80167 | 56.93586 |
| **Fuyang** | NA | 9.37518 | 8.22297 | 113.81267 | 61.56554 | 97.90849 |
| **Huainan** | NA | 4.83478 | 4.24058 | 58.6932 | 31.74935 | 50.49141 |
| **Bozhou** | NA | 9.07546 | 7.96008 | 110.17415 | 59.59733 | 94.77841 |
| **Hefei** | CA | 4.54262 | 3.98433 | 55.14648 | 29.8308 | 47.44031 |
| **Luan** | CA | 64.42757 | 4.65489 | 64.42757 | 34.85129 | 55.42446 |
| **Chuzhou** | CA | 90.28248 | 6.52291 | 90.28248 | 48.83718 | 77.6664 |
| **Huangshan** | SA | 5.81713 | 0.42029 | 5.81713 | 3.14671 | 5.00425 |
| **Chizhou** | SA | 13.42645 | 0.97006 | 13.42645 | 7.26287 | 11.55024 |
| **Xuancheng** | SA | 23.33727 | 1.68612 | 23.33727 | 12.624 | 20.07612 |
| **Maanshan** | SA | 20.54003 | 1.48402 | 20.54003 | 11.11087 | 17.66976 |
| **Wuhu** | SA | 26.57037 | 1.91971 | 26.57037 | 14.37291 | 22.85743 |
| **Tongling** | SA | 11.52341 | 0.83257 | 11.52341 | 6.23345 | 9.91313 |
| **Anqing** | SA | 40.7794 | 2.94631 | 40.7794 | 22.05911 | 35.08089 |

**Note: All data×10^5^ tons**

| **Geographical Regions** | **Utilization method** | **Average value** | **Standard error** | **ANOVA_F** | **ANOVA_p** | **KW_H** | **KW_p** | **Tukey** | **Significant annotation** |
| --- | --- | --- | --- | --- | --- | --- | --- | --- | --- |
| **NA** | Fertilization | 6.304 | 0.26875 | 7.028 | 0.008 | 8.215 | 0.016 | a | Significant |
| **CA** |  | 53.084 | 4.4775 | 7.028 | 0.008 | 8.215 | 0.016 | b | Significant |
| **SA** |  | 20.257 | 1.48125 | 7.028 | 0.008 | 8.215 | 0.016 | b | Significant |
| **NA** | Feed conversion | 6.142 | 0.2275 | 3.012 | 0.083 | 3.952 | 0.139 | a | Not Significant |
| **CA** |  | 5.054 | 0.23125 | 3.012 | 0.083 | 3.952 | 0.139 | a | Not Significant |
| **SA** |  | 1.552 | 0.13125 | 3.012 | 0.083 | 3.952 | 0.139 | a | Not Significant |
| **NA** | Energy conversion | 80.373 | 3.07 | 12.24 | 0.001 | 3.458 | 0.177 | a | Significant |
| **CA** |  | 69.952 | 3.11 | 12.24 | 0.001 | 3.458 | 0.177 | a | Significant |
| **SA** |  | 20.257 | 1.61125 | 12.24 | 0.001 | 3.458 | 0.177 | b | Significant |
| **NA** | Base materials | 43.477 | 1.78125 | 12.24 | 0.001 | 3.451 | 0.178 | a | Significant |
| **CA** |  | 37.839 | 1.7525 | 12.24 | 0.001 | 3.451 | 0.178 | a | Significant |
| **SA** |  | 10.976 | 0.85875 | 12.24 | 0.001 | 3.451 | 0.178 | b | Significant |
| **NA** | Raw materials | 69.141 | 2.76875 | 12.24 | 0.001 | 3.455 | 0.177 | a | Significant |
| **CA** |  | 59.51 | 2.61 | 12.24 | 0.001 | 3.455 | 0.177 | a | Significant |
| **SA** |  | 17.165 | 1.32 | 12.24 | 0.001 | 3.455 | 0.177 | b | Significant |
