# Supplementary material for: Exploring the contribution of straw utilization to carbon emission reduction in Anhui Province (China)
Source: PLoS One. 2026 May 27;21(5):e0349747. doi: 10.1371/journal.pone.0349747 (PMC13215477; doi:10.1371/journal.pone.0349747)
Supplement: S6 Table — (DOCX) [file pone.0349747.s006.docx]

**S6 Table Carbon neutrality intensity (CNI) characteristics of straw resource utilization in various regions**

| **City** | **Fertilization  CNI** | **Feed conversion CNI** | **Energy conversion**  **CNI** | **Base materials CNI** | **Raw materials CNI** |
| --- | --- | --- | --- | --- | --- |
| **Hefei** | 0.64 | 0.56 | 7.72 | 4.18 | 6.64 |
| **Huaibei** | 0.92 | 0.8 | 11.12 | 6.02 | 9.57 |
| **Bozhou** | 0.87 | 0.76 | 10.5 | 5.68 | 9.03 |
| **Suzhou** | 0.79 | 0.69 | 9.59 | 5.19 | 8.25 |
| **Bengbu** | 0.84 | 0.74 | 10.24 | 5.54 | 8.8 |
| **Fuyang** | 0.81 | 0.71 | 9.82 | 5.31 | 8.45 |
| **Huainan** | 0.82 | 0.72 | 10 | 5.41 | 8.6 |
| **Chuzhou** | 0.8 | 0.71 | 9.77 | 5.28 | 8.4 |
| **Luan** | 0.7 | 0.62 | 8.52 | 4.61 | 7.33 |
| **Maanshan** | 0.7 | 0.61 | 8.5 | 4.6 | 7.31 |
| **Wuhu** | 0.63 | 0.55 | 7.62 | 4.12 | 6.55 |
| **Xuancheng** | 0.65 | 0.57 | 7.93 | 4.29 | 6.82 |
| **Tongling** | 0.65 | 0.57 | 7.89 | 4.27 | 6.79 |
| **Chizhou** | 0.64 | 0.56 | 7.72 | 4.17 | 6.64 |
| **Anqing** | 0.6 | 0.53 | 7.28 | 3.94 | 6.26 |
| **Huangshan** | 0.48 | 0.43 | 5.88 | 3.18 | 5.06 |
